# Supplementary material for: Effectiveness of ultrasound-guided percutaneous transhepatic biliary drainage to reduce radiation exposure: A single-center experience
Source: PLoS One. 2022 Nov 4;17(11):e0277272. doi: 10.1371/journal.pone.0277272 (PMC9635691; doi:10.1371/journal.pone.0277272)
Supplement: Attachment — Submitted filename: Response to reviewer 3.docx [file pone.0277272.s002.docx]

**Response to Reviewer 3 Comments**

27 August, 2022

Dear reviewers and editorial staffs in *PLOS ONE*

We are sincerely grateful for your thorough consideration and scrutiny of our manuscript, “**Effectiveness of Ultrasound-guided Percutaneous Transhepatic Biliary Drainage to Reduce Radiation Exposure: A Single-Center Experience**”, control number PONE-D-22-13247. Through the accurate comments made by the reviewers, we better understand the critical issues in this paper. We have revised the manuscript according to the Reviewer’s suggestions. We hope that our revised manuscript will be considered and accepted for publication in the *PLOS ONE*. We acknowledge that the scientific and clinical quality of our manuscript was improved by the scrutinizing efforts of the reviewers and editors.

The changes within the revised manuscript were highlighted (in red). Point-by-point responses to the reviewers’ comments are provided below.

**Point 1:** Please focus more in the discussion section on the original elements of the study and on what you think this study brings in addition to what is already known.

**Response 1:** We appreciate reviewer’s comment. The purpose of our study was to evaluate the safety and effectiveness of US-guided PTBD. We found that US-guided PTBD showed a technical success rate of 100%, a clinical success rate of 94%, and a minor complication rate of 10%, thus US-guided PTBD was very safety and effectiveness. In addition, since PTBD procedure inevitably causes radiation exposure to both the operator and the patient, it is very important for the operator to recognize the problem of radiation exposure and make an effort to reduce it. Until recently, however, investigations of radiation exposure occurred during PTBD procedure are little. In this study, we especially focused on the radiation exposure (procedural data; fluoroscopy time and radiation dose reported as Dose Area Product, DAP), we found that the mean fluoroscopy time and radiation dose was significantly lower in patients with dilated bile ducts (group II) than those with non-dilated bile ducts (group I). In addition, in terms of the numbers of liver capsule punctures (intended as passage of the needle through the hepatic capsule) to gain the biliary tree and total procedural time, patients with non-dilated bile ducts showed significantly higher than patients with dilated bile ducts. In case of the reason of PTBD insertion (benign or malignant biliary stenosis) or the approach of PTBD insertion (right- and left-sided), there were no statistical significant differences. These results suggested that the most difficult and time-consuming reason for the operator who performing PTBD was directly related to the degree of bile duct dilatation, rather than reason for PTBD insertion (benign or malignant biliary stenosis/obstruction) nor the approach of PTBD insertion (right- or left-sided). Even though the number of liver capsule punctures, procedural time, fluoroscopy time and radiation dose were significantly higher in group I than group II, there were no statistical differences in terms of technical & clinical success rate and complication rate of US-guided PTBD between two groups. In addition, even in patients with non-dilated bile ducts undergoing an initial trial of biliary puncture under US guidance, the fluoroscopy time and the reported radiation dose were low, based on current study [19]. Perdersoli et al. [19] reported that the mean fluoroscopy time was 00:42:36 ± 00:35:39 h in nondilated bile ducts and the mean radiation dose was 18.6 ± 17.6 Gy cm² for non-dilated bile ducts. Whereas in our study, the mean fluoroscopy time was 317 ± 213.6 s and the mean radiation dose was 7.9 ± 4.1 Gy cm² for non-dilated bile ducts. Even if compared with procedural time in our study, the mean procedural time was 12.3 ±7.3 m in nondilated bile ducts. Thus, when performing PTBD in both patients with non-dilated bile ducts and dilated bile ducts, US-guided PTBD, initial trial of biliary puncture under US guidance, was found to be safe and effective technique significantly reducing radiation exposure. Therefore, we have updated discussion section as below.

**<Discussion>**

“PTBD is an effective treatment option for malignant or benign obstructive jaundice, especially in case of endoscopically inaccessible biliary tree or bile leak [2,3,4,5,6].”

“These discrepancies in fluoroscopy time and DAP compared with other studies are encouraging and probably attributed to active use of US in initial biliary puncture and active compliance with ALARA principle.”

“We observed that fluoroscopy time and DAP were significantly lower in group II than in group I, as expected. The results suggest that fluoroscopy time and radiation dose can be significantly lowered by performing US immediately before the procedure and biliary puncture under US guidance initially in patients with dilated bile duct. Giurazza et al. [12] reported 100% technical success of US-guided PTBD in 117 patients in a multicenter study. In their case series, 13.3% cases showed non-dilated IHD; the mean total fluoroscopy time was 570.4 s and the mean DAP was 37.25 Gy cm². In our study, 30% cases showed non-dilated IHD; the mean total fluoroscopy time was 202.6 s and the mean DAP was 5.2 Gy cm². We achieved not only a 100% technical success rate of PTBD under combined US and fluoroscopic guidance, but also remarkably with a lower fluoroscopy time and radiation dose. This discrepancy appeared to be due to an appropriate combination of US and fluoroscopy guidance to approach biliary puncture when US-guided puncture was not feasible immediately.”

“Even in patients with non-dilated bile ducts undergoing an initial trial of biliary puncture under US guidance, the fluoroscopy time and the reported radiation dose were low, based on current study [19]. Pedersoli et al. [19] reported that the mean fluoroscopy time was 00:42:36 ± 00:35:39 h and the mean radiation dose was 18.6 ± 17.6 Gy cm² for non-dilated bile ducts. Whereas in our study, the mean fluoroscopy time was 317 ± 213.6 s and the mean radiation dose was 7.9 ± 4.1 Gy cm² for non-dilated bile ducts. Even if compared with procedural time in our study, the mean procedural time was 12.3 ±7.3 m in nondilated bile ducts. Thus, US-guided PTBD was found to be a safe and effective technique significantly reducing fluoroscopy time and radiation dose.”

“There were no significant differences in the number of liver capsule punctures, procedural time, fluoroscopy time, DAP, and complication rates between right- and left-sided PTBD.”

“In cases with benign biliary stenosis/obstruction, the IHD was not markedly dilated compared with malignant biliary stenosis/obstruction. This results are well consistent with previous study [26]. Procedural time, fluoroscopy time and DAP in cases with malignant biliary stenosis/obstruction tended to be lower than those in cases with benign biliary stenosis/obstruction, however, there were no statistical significance. In addition, the number of liver capsule punctures was higher in group I than in group II with statistically significance, meaning the number of liver capsule puncture was correlated with IHD dilatation. These results suggested that the most difficult and time-consuming reason for the operator performing PTBD was directly related to the degree of IHD dilatation, rather than the reason for PTBD insertion (benign or malignant stenosis/obstruction) nor the approach of PTBD insertion (right- or left-sided).”
